# Imaging of *Streptomyces coelicolor* A3(2) with Reduced Autofluorescence Reveals a Novel Stage of FtsZ Localization

**DOI:** 10.1371/journal.pone.0004242

**Published:** 2009-01-21

**Authors:** Joost Willemse, Gilles P. van Wezel

**Affiliations:** Microbial Development, Department of Molecular Genetics, Leiden Institute of Chemistry, Leiden University, Leiden, The Netherlands; Charité-Universitätsmedizin Berlin, Germany

## Abstract

Imaging of low abundance proteins in time and space by fluorescence microscopy is typically hampered by host-cell autofluorescence. Streptomycetes are an important model system for the study of bacterial development, and undergo multiple synchronous cell division during the sporulation stage. To analyse this phenomenon in detail, fluorescence microscopy, and in particular also the recently published novel live imaging techniques, require optimal signal to noise ratios. Here we describe the development of a novel derivative of *Streptomyces coelicolor* A3(2) with strongly reduced autofluorescence, allowing the imaging of fluorescently labelled proteins at significantly higher resolution. The enhanced image detail provided novel localization information for the cell division protein FtsZ, demonstrating a new developmental stage where multiple FtsZ foci accumulate at the septal plane. This suggests that multiple foci are sequentially produced, ultimately connecting to form the complete Z ring. The enhanced imaging properties are an important step forward for the confocal and live imaging of less abundant proteins and for the use of lower intensity fluorophores in streptomycetes.

## Introduction

The discovery and cloning of the *gfp* gene of *Aequorea victoria* [1], [2] and the subsequent creation of many coloured derivatives [3] have provided cell biologists with a set of powerful tools to analyze the expression and localization in time and space of proteins of interest in almost all organisms and research fields [4]. Widely applied techniques study the localization [5], [6], order of appearance [7], [8], dynamic properties [9]–[11] and interaction partners of the proteins of interest [12], [13]. Localization studies through time and space of bacterial cell division proteins have created significant insight into the underlying mechanism [14], [15]. Disturbingly, autofluorescence of the organism studied severely limits the application of fluorescent imaging, especially when using low abundant proteins or weaker fluorophores (such as RFP or CFP). Fluorescence imaging benefits from optimized signal to noise ratios, and background fluorescence and other noise frustrate the study of low abundant proteins at natural expression levels.

*Streptomyces coelicolor* is a Gram-positive soil bacterium with an unusually complex mycelial life cycle. *Streptomyces* growth on solid media starts with the germination of a single spore that develops into a complex vegetative mycelium of branching hyphae [16]. Environmental signals such as nutrient depletion result in the development of initially aseptate aerial hyphae while part of the vegetative mycelium lyses [17]–[19]. Eventually, the aerial hyphae are dissected into spores by specialized sporulation septa, producing chains of connected uninucleoid spores [20]. While in most bacteria a single septum is formed that forms the cleavage furrow dividing the mother cell into the daughter cells, during sporulation of *Streptomyces* many septa are simultaneously produced to form long chains of spores [21], [22]. Several novel protein families have been uncovered that play a role in the control of septum formation, the localization of which is studied by advanced fluorescence microscopy [23]–[26]. Relatively high background fluorescence in addition to the fact that several of the developmentally essential proteins are expressed at a low level makes fluorescence microscopic analysis of such proteins difficult to perform. Since altering the expression level of target proteins generally is not an option, we created a bacterial strain with reduced autofluorescence, and used this to provide novel information on the mechanism of sporulation-specific cell division in the important model organism *Streptomyces coelicolor* A3(2).

## Results and Discussion

**Figure 1:**
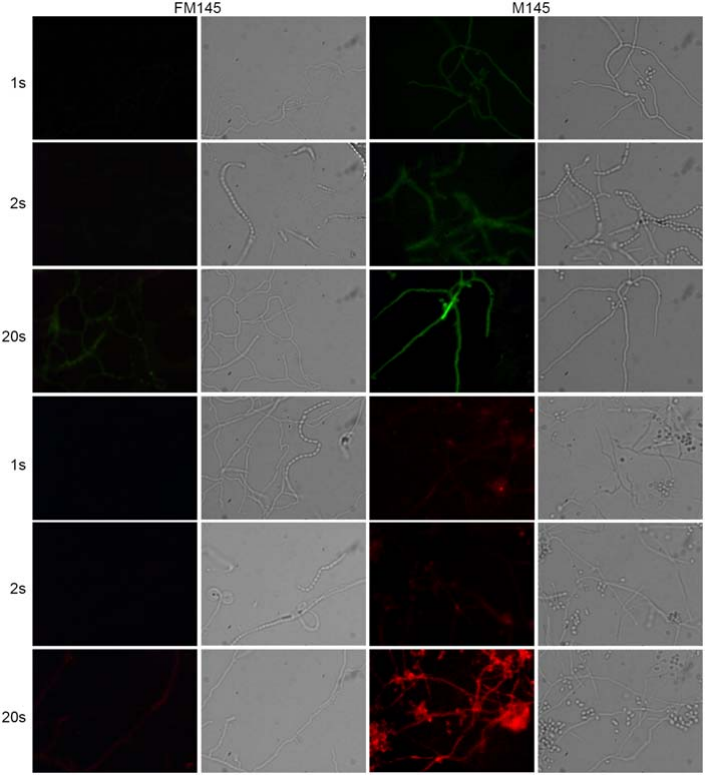
Autofluorescence of *S. coelicolor* M145 and FM145. Green autofluorescence (top two rows; 470–490 nm excitation and 515 long pass detection) and red autofluorescence (bottom two rows; 530–550 nm excitation and 590 long pass detection) in *S. coelicolor* FM145 (1^st^ column) and its parent M145 (3^rd^ column). Corresponding light images are presented in the 2^nd^ and 4^th^ columns, respectively. Exposure times are shown on the left side of the image. Autofluorescence is clearly reduced in FM145.

By streaking single colonies of *S. coelicolor* M145 on SFM (soy flour mannitol) agar plates [27] and subsequently selecting colonies with the lowest level of autofluorescence, we obtained several strains with reduced autofluorescence. These were passed through an initial characterization of growth rate and morphogenesis. Derivatives with wild-type appearance were then selected. One strain, designated FM145, was selected for intensive further scrutiny. FM145 displayed about threefold decrease (2.9±0.3 based on at least 100 hyphae) in autofluorescence in the green channel (Figure 1a) and more than fivefold (6.9±1.3) in the red channel (Figure 1b).

**Figure 2:**
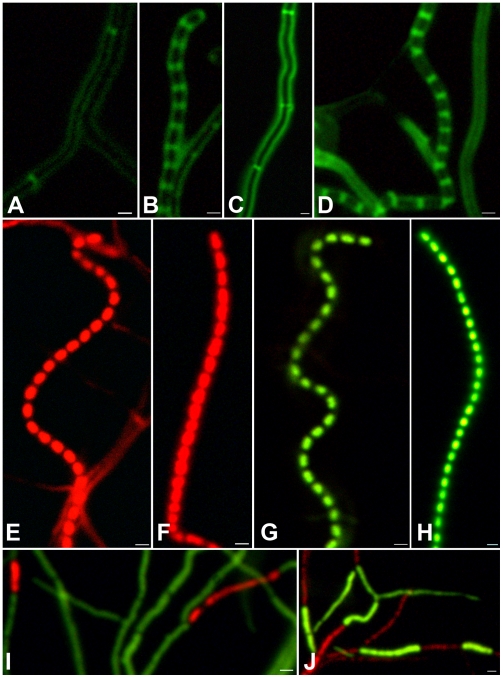
*S. coelicolor* FM145 has a wild-type phenotype. Fluorescence micrographs of *S. coelicolor* FM145 (a–b, e, g, and i) and M145 (c–d, f, h, j), grown against microscope cover slips on SFM agar plates for 64 hr. Samples were stained with FITC-WGA (a–d), with propidium iodide (red; e–f) or with a mixture of Cyto 82 (green) and propidium Iodide (g–j). The images present vegetative hyphae with cross-walls (a, c; signal to noise is 3.2∶1 and 1.4∶1, respectively), sporogenic aerial hyphae with ladders of septa (b, d; signal to noise ratio of 2.5∶1 and 1.9∶1 respectively), or spore chains (e–h) and vegetative hyphae (i–j) None of these comparisons revealed significant phenotypic differences between FM145 and its parent. Signal to noise ratios could not be determined for 1e–j as staining of the entire hyphae prevented determination of background fluorescence. The exposure times were 1 second (2a–d) or 200 ms (2e–j).

The new derivative FM145 was compared extensively to its parent *S. coelicolor* M145 to rule out defects in growth or morphogenesis. Initial tests, comparing phenotypes on plates and by stereo and phase-contrast microscopy, revealed no differences between these strains. Both strains grew with similar growth rates and the development of aerial hyphae, grey-pigmented spores and the production of pigmented antibiotics was initiated at the same time (data not shown). There was no statistically relevant difference in the branching frequencies (once per 8.9±0.9 µm for FM145 and once per 7.6±0.7 µm for M145) or in the average spore chain lengths (26±3 spores for FM145 and 28±2 spores for M145). For a more detailed analysis and considering its use in fluorescence microscopy, FM145 and its parent M145 were analyzed using three different stains. FITC-labelled wheat germ agglutinin (FITC-WGA) was used to visualize newly synthesized peptidoglycan and in particular the septa; propidium iodide (PI) was used to visualize DNA and cell viability was imaged by staining with a combination of Cyto 82 and PI dyes [28]. For all stains we obtained significantly better signal to noise ratios for FM145 than for M145, but no relevant biological differences were observed between the strains. FITC-WGA staining showed that the spacing between the septa in vegetative hyphae (Figure 2a,c) and between the sporulation septa in sporogenic aerial hyphae (Figure 2b,d) was very similar, while propidium iodide (PI) staining showed that all spores contained similar amounts of DNA (Figure 2e,f). The use of a combination of Cyto 82 as viable cell stain (green) and PI for identification of damaged or dying cells (red), again demonstrated a very similar pattern of lysing cells and viable cells in vegetative hyphae of M145 and FM145, again illustrating the wild-type growth behaviour of FM145 (Figure 2i–j).

**Figure 3:**
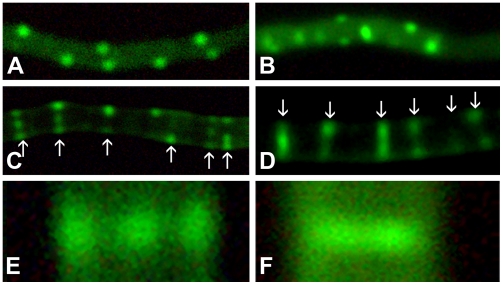
Localization of FtsZ-eGFP. The localization of FtsZ-eGFP in FM145 (a,c,e) and M145 (b,d,f) was analysed in sporogenic aerial hyphae. For illustration purposes, identical single-pass background correction was performed for both images. Localization in foci (a–b) slowly changes to a combination of foci and completed Z rings (c–d), the arrows here indicate the deconvoluted septa shown in figure 4. A close up of FtsZ distribution on a single septum shows three foci in FM145 (e) whereas no clear foci are distinguishable in the parental strain M145 (f). All images were made with 1 second exposure time. Note the strongly reduced background (3a) and the much sharper foci (3c,e) in FM145 as compared to its parent M145 (images 3b and 3d, f, respectively).

To analyze the possible improvement in detail and strength of the localization signals (*i.e.* foci) we transformed both strains with a construct expressing a translational fusion of FtsZ and eGFP behind the wild type FtsZ promoter, making use of the pKF41 vector described previously [5]. Both images were background-corrected with an exposure time of one second. This clearly demonstrated the improved signal to noise ratio of FM145 (Figure 3a,c) over its parent M145 (Figure 3b,d), with major reduction in autofluorescence in the new derivative. Ladders of Z rings were clearly visible in both strains. As described previously [5] several localization patterns are observed in the aerial hyphae. Initially a dispersed pattern in sporogenic aerial hyphae marking the earliest stages of differentiation, followed by patches and filaments of irregular lengths (Figure 3a–b), finally culminating in the typical ladder-like pattern corresponding to the future septa. Interestingly, the reduced autofluorescence allowed visualization of an additional fourth pattern corresponding to a new stage of FtsZ localization that could not be identified before due to the lower resolution; this stage occurred prior to completion of the Z rings, with differential distribution of FtsZ over the individual septa observed as multiple foci (Figure 3c and e). This pattern was observed just before the final ladders appeared and apparently highlights a transition phase between regular spaced FtsZ foci to complete Z rings.

**Figure 4:**
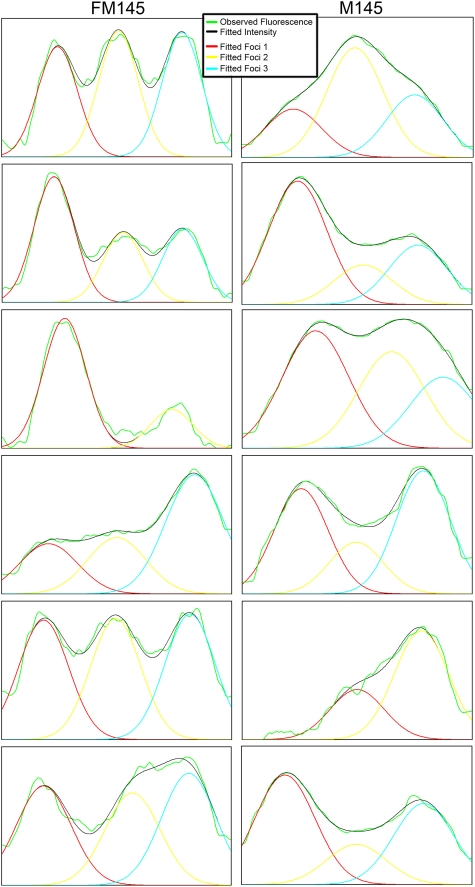
Deconvolution of FtsZ fluorescence. Images show the fluorescence-intensity patterns and their deconvoluted single foci indicated by the arrows in Figure 3cd. Order of images: graphs from top to bottom correspond to images from left to right. Left column, FM145 (corresponding to the septa in Figure 3c); right column, M145 (corresponding to the septa in Figure 3d).

To confirm this additional localization pattern of FtsZ, deconvolution studies were performed on the fluorescence intensity plots of a number of individual septa. These studies revealed that several fluorescence-intensity patterns are in fact built up of multiple foci, although the foci on a single septum cannot be visually distinguished in M145. All septa shown in Figures 3c and 3d have been analysed and the deconvolved intensity profiles show that the FtsZ fluorescence in most septa at this stage actually are composed of three distinct foci (Figure 4), not only in FM145 but also in M145. Additionally we have analysed the fluorescence-intensity patterns in 40 randomly selected sporulation septa of a strain co-expressing SsgA-GFP and FtsZ-mCherry. In this experiment SsgA is used as a control for the deconvolution studies; SsgA forms multiple foci in sporulating aerial hyphae but with a localization pattern that is distinct from that of FtsZ, lacking the typical ladder-like fluorescence pattern [26]. FtsZ localised as two foci, three foci or as a continuous ring (*i.e.* more than three foci). In contrast, analysing the SsgA localization patterns in the same images revealed localization into one, two or three foci at the septal planes, but not as a ring. These experiments demonstrate that the lack of single foci and the presence of complete rings were characteristic of FtsZ localization. According to Abbe's resolution limit the maximum resolution for light microscopy is defined by the excitation wavelength and the numerical aperture of the objective used. For these studies the resolution is therefore limited to around 250 nm. Therefore the visualization of three foci on a septum is the theoretical maximum dictated by the width of the hyphae (around 800 nm) and the resolution limit. Based on the demonstrated multi-focal stage we propose that Z-ring assembly on the septum is a step-wise process, where FtsZ foci are sequentially formed at the septal plane, eventually forming the complete Z ring, rather than that the Z ring is produced from two gradually expanding spots. Recently developed resolution-breaking microscopy techniques [29], [30] can help determine whether there are indeed subsequent stages of Z-ring assembly. This is a clear example of the type of additional information that may be obtained when using a strain with reduced background fluorescence, thus revealing novel biological data.

The nature of the reduction of autofluorescence in FM145 is yet unknown. However, as shown above, the comparison with the parent did not reveal significant changes in the morphological development of this new strain. Repetitive non-selective streaking of FM145 readily results in strains with increased autofluorescence, indicating that the change in FM145 is most likely due to the reduced synthesis of a fluorescent compound rather than to a disruptive mutation (not shown).

In conclusion, our experiments demonstrate that the new derivative FM145 has significantly reduced autofluorescence, but is otherwise highly similar to its parent *S. coelicolor* M145. From the biological perspective, the differences in signal intensities allowed us to demonstrate differential localization of FtsZ-eGFP at the septa in the later stages of development of sporogenic hyphae of *S. coelicolor*. From the technical point of view, the improved detail allows better imaging of weaker signals. Complementation studies are required to identify the molecular basis of the autofluorescence, which would allow the targeted reduction of autofluorescence in other micro-organisms. The availability of a strain with reduced autofluorescence and an otherwise wild-type phenotype offers new possibilities for live-imaging techniques [25] and for fluorescence microscopic studies on less abundant proteins in the important model organism *Streptomyces*.

## Materials and Methods

### Growth conditions and media

*Streptomyces coelicolor* A3(2) M145 was obtained from the John Innes centre strain collection and served as a parent for the low autofluorescence screen carried out solely on SFM agar plates at 30°C [27].

### Fluorescent imaging

All images were obtained with an Olympus bh-2 upright fluorescence microscope making use of a mounted Colorview IIIu (Soft Imaging Systems) at a resolution of 17.5 nm/pixel (2576×1932 pixels) using a 100× Dapo olympus N.A. 1.3 oil objective. The green fluorescent images were created using 470–490 nm excitation and 515 long pass detection, for the red channel 530–550 nm excitation and 590 long pass detection was used. All images were background corrected setting the signal outside the hyphae to zero to obtain a sufficiently dark background. These corrections were made using Adobe Photoshop CS (v 8.0)

### Deconvolution

Using ImageJ 1.37c a plot profile of the septa was obtained for all fluorophores (eGFP, mCherry). The profile plots were obtained with a line width of 200 nm. Subsequently, the obtained raw data were analysed using Peakfit v4.12 for windows. Deconvolution was performed with the automatic deconvolution section of the program in which we set the expected full-width half maximum based on the theoretical resolution as determined by Abbe's formula 0.61λ/N.A. (λ is the excitation wavelength, N.A. is the numerical aperture of the objective used). The resulting fits were used to determine the amount of foci in each fluorescent profile.
